# Arterial Infusion Chemotherapy for Neoplastic Esophagogastric Anastomotic Strictures After Esophagectomy

**DOI:** 10.3389/fonc.2021.668593

**Published:** 2021-05-26

**Authors:** Pengfei Xie, Meipan Yin, Wei He, Yaozhen Ma, Chunxia Li, Zhen Li, Xiaobing Li, Shuai Wang, Gang Wu

**Affiliations:** ^1^ Department of Interventional Radiology, The First Affiliated Hospital of Zhengzhou University, Zhengzhou, China; ^2^ Oncology Department, The First Affiliated Hospital of Zhengzhou University, Zhengzhou, China

**Keywords:** esophageal cancer, esophagogastric anastomotic stenosis, arterial infusion chemotherapy, esophagectomy, interventional radiology

## Abstract

**Background:**

Neoplastic esophagogastric anastomotic strictures after resection of esophageal cancer is a very difficult problem in clinical practice. We aim at to investigate the safety and feasibility of arterial infusion chemotherapy in treatment of neoplastic esophagogastric anastomotic strictures after esophagectomy.

**Methods:**

From October 2014 to December 2019, 50 patients with Neoplastic esophagogastric anastomotic strictures after resection of esophageal cancer were assessed retrospectively. Preoperative dysphagia was grade III in 34 cases and grade IV in 16 cases. Thirty-eight patients had different degrees of dyspnea before surgery Twenty-five patients had intolerable (grade IV) dyspnea and airway stenting was undertaken before surgery. Thirteen patients had tolerable dyspnea that did not require airway stenting, and preoperative dyspnea was grade III.

**Results:**

All patients were successfully treated with arterial infusion chemotherapy, no paraplegia or death occurred. The dysphagia grade of 50 patients after AIC was compared: one case had grade I, 40 cases had grade II, and nine cases had grade III. Thirteen patients had tolerable dyspnea that did not necessitate airway stenting. Dyspnea was classified as grade I in five cases and grade II in eight cases. After 1–3 courses of AIC, 50 patients were followed up for a complete response (eight cases), partial response (28) and stable disease (14 cases). Total objective effective rate (complete response+ partial response) and disease control rate(complete response + partial response + stable disease)were 72.0% and 100.0%, respectively. The median duration of follow-up was 8.5 months. One-year survival was 46.0%.

**Conclusion:**

Arterial infusion chemotherapy is safe and efficacious treatment for Neoplastic esophagogastric anastomotic strictures after esophagectomy.

## Introduction

Neoplastic esophagogastric anastomotic strictures (NEAS) after esophagectomy often manifest as dysphagia, which seriously affects the quality of life and survival of patients (1–3). Balloon dilatation is common treatment for benign stricture of an esophagogastric anastomosis (4, 5), but it is not suitable for NEAS (6, 7). The treatment methods for NEAS after esophagectomy include stent implantation into the esophagus, resection, radiotherapy, chemotherapy, and placement of a radioactive nutrition tube (8–11).

Arterial infusion chemotherapy (AIC) for esophageal cancer can greatly increase the drug concentration in the tumor, which can reduce the tumor mass rapidly and reduce airway compression and tumor invasion. AIC for esophageal cancer has definite curative effect and little side effect (12, 13). There are changes in the anatomical structure of the feeding arteries after surgery for esophageal cancer. We wished to investigate the safety and efficacy of AIC against NEAS after esophagectomy.

## Materials and Methods

### Ethical Approval of the Study Protocol

This study protocol was approved by the ethics investigation committee of The First Affiliated Hospital of Zhengzhou University. Ethical approval code: SS-2018-22. Written informed consent was obtained from each patient during questionnaire administration for the collection and analysis of applicable clinical data.

### Inclusion and Exclusion Criteria

The inclusion criteria were: (i) NEAS confirmed by imaging and pathology findings; (ii) patients were treated with interventional chemotherapy; (iii) Age >18 yr and Age <85yr, gender unlimited; (iv) all patients refused the second operation. The exclusion criteria were: (i) a benign cicatricial stricture of an esophagogastric anastomosis; (ii) AIC was not used to treat NEAS; (iii) AIC for unresectable esophageal cancer.

### Data Source

We retrospectively analyzed the data of patients with NEAS after resection of an esophageal tumor treated by AIC in the Interventional Therapy Center of The First Affiliated Hospital of Zhengzhou University from October 2014 to December 2019. These data comprised medical records, imaging findings, interventional procedures, and follow-up information.

### AIC

#### Preoperative Preparation

Routine hematology, liver/kidney function, electrolytes, electrocardiography and contrast-enhanced computed tomography (CT) were completed preoperatively to evaluate the physical and nutritional status of patients.

Patients with grade-IV dysphagia were treated with an interventional procedure under fluoroscopy. Placement of a nasojejunal nutrition tube and AIC was done 5–7 days later.

**Figure 1:**
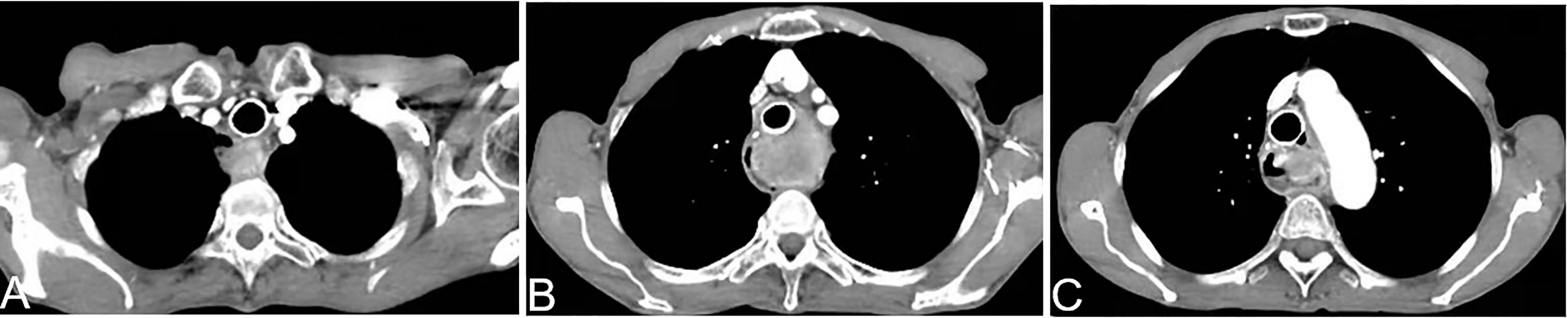
A 69-year-old woman presented with dyspnea of 1-month duration >5 years after esophagectomy. Airway stenting was done 1 week before arterial infusion chemotherapy. The airway lumen was unobstructed. A chest CT scan shows that the soft tissue of the esophagogastric anastomosis was enhanced obviously **(A–C)**.

Patients with an airway stricture and dyspnea due to compression/invasion by the tumor were evaluated. If the patient could lie down under oxygen inhalation, stent placement to relieve an airway stricture was not indicated. If the patient had severe dyspnea and could not lie down under oxygen inhalation, a stent was implanted in the airway to relieve dyspnea, and AIC was undertaken 5–7 days later (**Figure 1**).

#### Procedure

**Figure 2:**
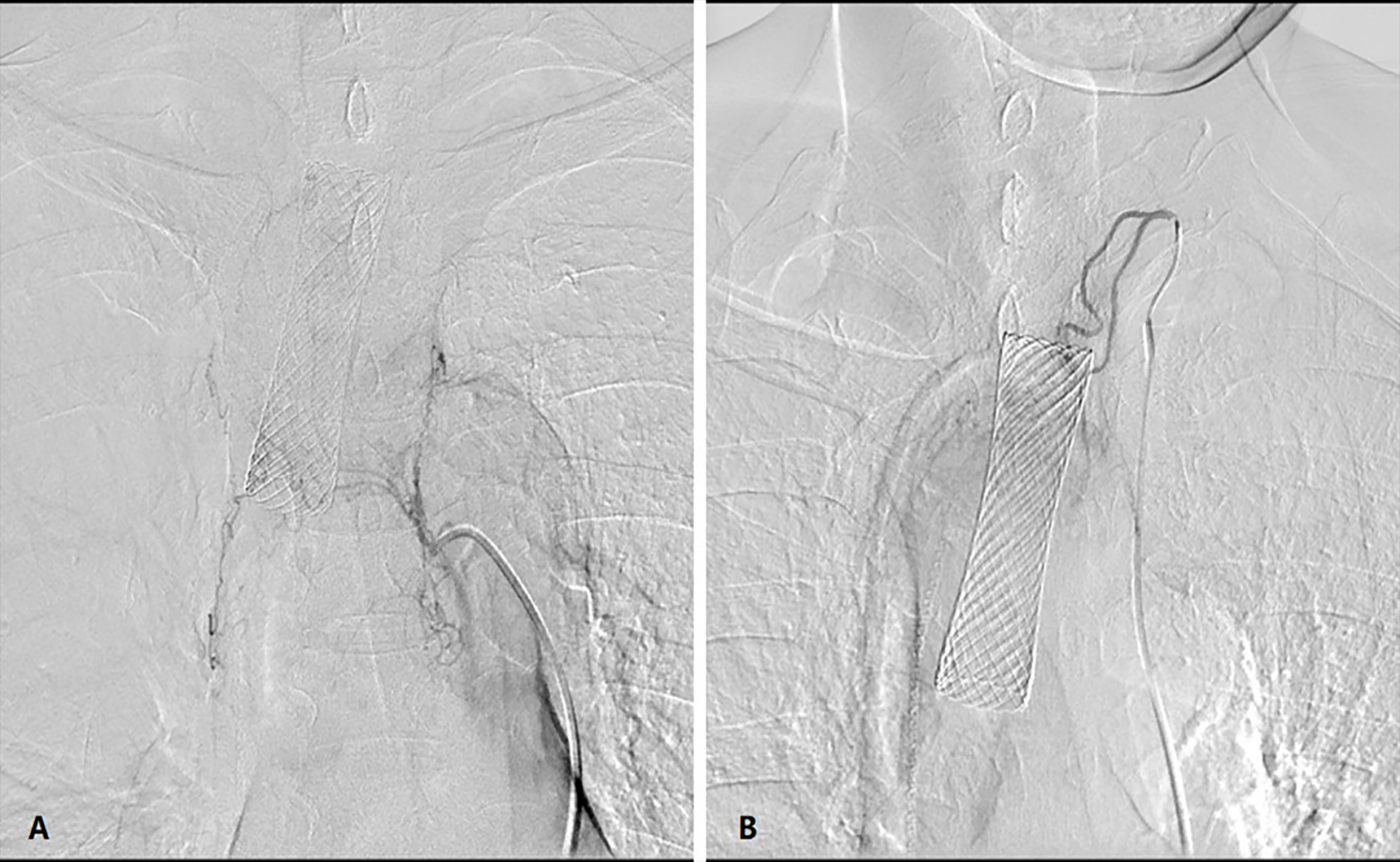
DSA showed that the **(A)** left inferior thyroid artery and **(B)** bronchial artery participated in the blood supply to the tumor.

The patient was supine on a table designed for digital subtraction angiography (DSA). The procedure is usually performed by an attending doctor and a fellow. In the conscious state, puncture of the femoral artery was undertaken, and a 5-F arterial sheath inserted. A 5-F “cobra” catheter or vertebral-artery catheter was introduced through the arterial sheath to identify the corresponding feeding arteries of the lesion. Usually, neoplastic lesions of the esophagogastric anastomosis are supplied by the inferior thyroid artery, bronchial artery, right gastroepiploic artery or right gastric artery. According to the body surface area and physical condition of the patients, in general, adriamycin (30–50 mg), oxaliplatin (100 mg) or raltitrexed (4 mg) are used. The appropriate compatible solution of each chemotherapy drug was diluted in 150 mL. The dose of chemotherapy drugs was allocated according to the blood supply of the target vessels within the lesion. The duration of perfusion of each drug was maintained at 15–20 min (**Figure 2**).

#### Postoperative Care

**Figure 3:**
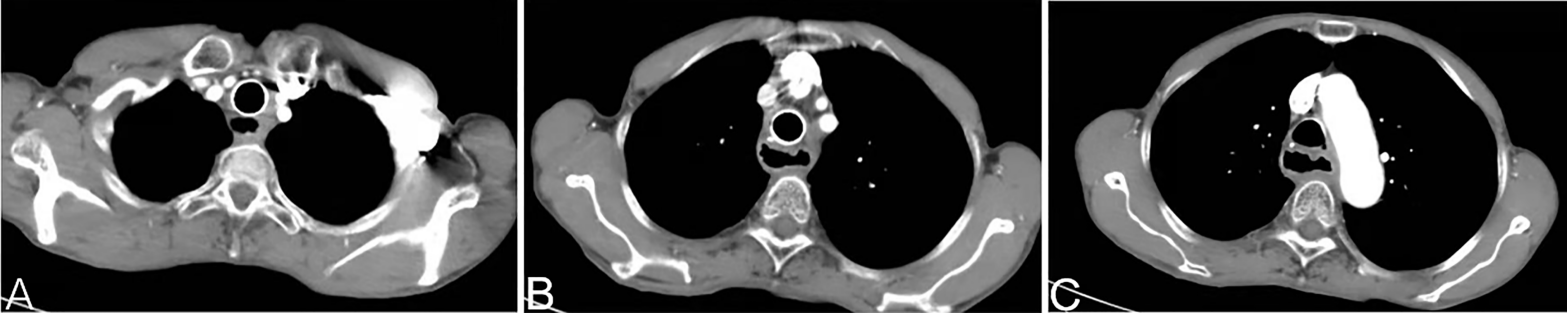
A chest CT at 1 month after arterial infusion chemotherapy shows that the soft-tissue shadow of the esophagogastric anastomosis to be reduced significantly **(A–C)**.

Patients were given anti-emetic agents, anti-acid agents and hydrated. Routine hematology, liver/kidney function, electrolytes and other indicators were monitored for 7 days after surgery. Leukocytes and platelets were administered if the counts for leukocytes and platelets were low. One month after procedure, CT of the chest was done to evaluate the curative effect (**Figure 3**).

### Evaluation Criteria for Clinical Efficacy and Adverse Reactions

Dysphagia was assessed using Stooler grading criteria (14). Patients with an esophageal fistula and severe stricture of an esophagogastric anastomosis were treated with a nasojejunal nutrition tube. The degree of stricture was determined by DSA.

According to the classification standard for dyspnea set by the American Thoracic Association (ATA), all patients with a stricture in their airways were evaluated for improvement of respiratory function 1–7 days before and after stent implantation in the airways, or after AIC for esophageal cancer.

Clinical staging of all patients before and after treatment was evaluated according to the criteria for clinical staging set by the American Joint Committee on Cancer (15). The decrease of clinical stage after AIC represents the better effect of AIC. The clinical efficacy of drugs used to treat esophageal cancer was evaluated according to the complete response (CR), partial response (PR) and whether the patient had stable disease (SD) or progressive disease (PD) (16, 17). CR+PR was defined as the objective response rate (ORR). Disease control rate (DCR) was defined as CR+ PR + SD.

If the curative effect of the lesion reached a CR, then conversion to radiotherapy was indicated. If the curative effect of the lesion reached a PR or SD, pulse perfusion chemotherapy was undertaken. If the lesion was evaluated as PD, other types of palliative treatment were indicated.

Adverse reactions to the chemotherapy drugs, changes in tumor size, and laboratory test results were recorded. The duration of survival was documented. The toxicity and side-effects of chemotherapy drugs were evaluated according to National Cancer Institute Common Toxicity criteria and classification of anticancer-drug toxicity (0–IV).

### Statistical Analyses

Data were analyzed using Prism 5 (GraphPad, San Diego, CA, USA). An independent two-sample *t*-test was used to compare quantitative data. Data are the mean ± SD. The Student’s *t*-test was used to compare continuous variables. The chi-square test was employed to compare categorical data. P < 0.05 was considered significant.

## Results

### General Information

Fifty patients with NEAS after esophagectomy (32 men and 18 women; mean age, 63.6 ± 8.8 years) formed the study cohort. Of these 50 cases, two had hypertension, three had diabetes mellitus (DM), and one had hypertension and DM. Thirty-eight cases were complicated with different degrees of airway stricture: tracheal stricture (30 cases), carina-region stricture (one), stricture of the right-main bronchus (one) and stricture of the left-main bronchus (six; including three cases of stricture of the left-main bronchus combined with atelectasis). Among the 50 cases, 10 cases were complicated with an esophageal fistula (eight cases of an esophagotracheal fistula and two cases of esophagogastric anastomotic fistula) (**Table 1**).

**Table 1:** Patient characteristics.

	Number of cases
Patients	50
Males	32 (64.0%)
Mean age, years	63.6 ± 8.8
Comorbidity	
Hypertension	2
Diabetes mellitus	3
Location of airway stricture	
Carina of main trachea	1 (2.6%)
Right-main bronchus	1 (2.6%)
Left-main bronchus	6 (15.8%)
Main trachea	30 (79.0%)
Types of esophageal fistula
Esophageal airway	8 (80.0%)
Esophageal neck	2 (20.0%)

The basic information of all patients before and after esophagectomy and before recurrence is shown in **Table 2**.

**Table 2:** Patient characteristics of before and after surgery.

	Number of cases
Clinical stage of primary tumor
T2	10 (20.0%)
T3	34 (68.0%)
T4a	6 (12.0%)
Tumor location
Upper esophagus	8 (16.0%)
Middle esophagus	28 (56.0%)
Lower esophagus	14 (28.0%)
Tumor histotype
Squamous cell carcinoma	50 (100.0%)
Tumor differentiation	27 (54.0%)
Medium differentiation	23 (46.0%)
Low differentiation	21 (42.0%)
Induction chemotherapy
Induction radiotherapy	18 (36.0%)
Adjuvant chemotherapy	31 (62.0%)
Type of esophagectomy
Radical esophagectomy	50 (100.0%)
Technique of anastomosis
Stapler	50 (100.0%)
Type of lymph nodal dissection
Two Field	35 (70.0%)
There Field	15 (30.0%)
Anastomotic location
Cervical anastomosis	37 (74.0%)
Mediastina anastomosis	13 (26.0%)
Median DFS(Month)	13

### Treatment

Among the 38 patients with different degrees of an airway stricture before procedure, 13 patients had tolerable dyspnea without the requirement of an airway stent, and 25 patients with a severe stricture of the airways who could not tolerate AIC were treated with an airway stent. Among these 25 patients, four cases underwent implantation with a Y-type metal stent, and 21 cases had a tubular metal stent. Eleven patients with grade-IV dysphagia due to a neoplastic anastomotic stricture were treated with a nasal nutrition tube under fluoroscopy guidance. Ten patients with NEAS (including 5 cases of IV dysphagia) and an esophageal fistula had conservative treatment (e.g., nutrition-tube placement) and one patient with an esophageal fistula was treated with a covered airway stent.

In 50 patients, the feeding artery of the tumor was identified and perfused with a chemotherapy drug. For each patient, 1–4 feeding arteries were perfused, including the bilateral inferior thyroid artery (11 cases), unilateral inferior thyroid artery (six), bilateral bronchial artery (16), unilateral bronchial artery (30), right gastroepiploic artery (27), thyroid carotid artery (five) and right gastric artery (three). When the 5F catheter could not enter the tumor feeding artery, the microcatheter was used to superselect the tumor feeding artery. When intercostal artery and bronchial artery are involved, microcatheter should be used to bypass the potential spinal artery branch and be as close as possible to the tumor feeding artery. Intraoperatively, a microcatheter was used for super-selective intubation 63 times Thirty-seven patients received one course of AIC, 10 patients received two courses of AIC, and three patients received three courses of AIC.

### Evaluation of Clinical Efficacy

Fifty patients who underwent esophagectomy had different degrees of stricture of an esophagogastric anastomosis before AIC: 34 cases had grade III and 16 cases had grade IV. According to Stooler grading criteria for dysphagia classification, after AIC, one patient had grade I, 40 cases had grade II, and nine cases had grade III. The degree of stricture of the esophagogastric anastomosis was relieved to different degrees after AIC. Eight of the 11 patients who underwent nasogastric intubation due to an esophagogastric anastomotic stricture had the nutrition tube removed and oral feeding was initiated after 3 weeks of AIC. Among them, three cases continued to have grade-III dysphagia. Twenty-five patients had dyspnea before surgery. According to the ATA classification of dyspnea, all 25 patients were grade IV before stenting. After tracheal stenting, oxygen saturation was >95% without oxygen inhalation, and 11 cases had grade-II and 14 cases had grade-III dyspnea. Thirteen patients with an airway stricture could tolerate dyspnea without implantation of an airway stent. According to the ATA classification of dyspnea, all 13 patients had grade-III dyspnea preoperatively. One week after surgery, oxygen saturation was >95% without oxygen inhalation. Among them, five cases had grade-I and eight cases had grade-II dyspnea. In one patient with a fistula in the neck portion of the esophagus, closure was achieved 3 weeks after placement of a nasojejunal nutrition tube. One patient with an esophagotracheal fistula was treated with a covered airway stent and nutrition tube, and the fistula healed completely after one course of AIC.

The clinical stage of all patients before treatment was T3 (six cases) and T4b (44). After 1–3 courses of treatment, 50 patients were followed up, and the clinical stage was T1 (four cases), T2 (12), T3 (16) and T4b (18 cases). After treatment, the clinical stage decreased significantly (**Table 3**).

**Table 3:** Clinical classification before and after arterial infusion chemotherapy.

Classification	Before the first treatment	After the first course	Before the second treatment	After the second course	Before the third treatment	After the third course
n	50	50	13	13	3	3
T1	0	2*	0	1	0	1
T2	0	9**	1	4	0	1
T3	6	21***	9	6	2	0
T4b	44	18***	3	2	1	1

***p < 0.0001, **p < 0.005, *p < 0.05.

After the first course of AIC, a CR was noted in four cases, PR in 31 cases and SD in 15 patients and the ORR was 70.0%. Thirteen patients received a second course of AIC for esophageal cancer. After the second course, a CR was noted in two cases, PR in 10 patients and SD in one case, and the ORR was 92.3%. Three patients received a third course of AIC for esophageal cancer, after which a CR was noted in two cases, SD in one patient, and the ORR was 66.7%. After 1–3 courses of treatment, a CR was documented in eight cases, PR in 28 patients, and SD in 14 cases, and the ORR was 72.0%, and the DCR was 100% (**Table 4**).

**Table 4:** Clinical efficacy after arterial infusion chemotherapy.

Classification	After the first course	After the second course	After the third course	Follow-up
n	50	13	3	50
Complete response	4 (8.0%)	2 (15.4%)	2 (66.7%)	8 (16.0%)
Partial response	31 (62.0%)	10 (77.0%)	0	28 (56.0%)
Stable disease	15 (30.0%)	1 (7.7%)	1 (33.3%)	14 (28.0%)
Overall response rate	35 (70.0%)	12 (92.3%)	2 (66.7%)	36 (72.0%)
Disease control rate	50 (100.0%)	13 (100.0%)	3 (100.0%)	50 (100.0%)

Overall response rate = complete response + partial response.

Disease control rate = complete response + partial response + stable disease.

### Complications

Adverse reactions of grade I–III occurred after AIC for esophageal cancer (**Table 5**). Nausea and vomiting (42.0%) were noted in 21 cases, thrombocytopenia was documented in seven patients (14.0%), fever in three cases (6.0%), and leukopenia in eight cases (16.0%). These and other common adverse reactions were relieved in a short time after symptomatic treatment.

**Table 5:** Adverse reactions after arterial infusion chemotherapy.

	I	II	III
Lowering of white blood cell count	6	1	1
Thrombocytopenia	5	2	0
Vomiting	9	10	1
Fever	1	2	0

### Follow-Up and Survival

During follow-up, the median duration of survival was 8.5 months. Twenty five patients died of systemic organ failure at the end of the tumor. The duration of survival (in months) of seven patients was 12.0 ± 1.8, and that of five patients was 24.0 ± 1.2. Thirteen patients died of tumor-related respiratory failure, and five patients survived for 12.0 ± 2.1 months. Seven patients died of gastrointestinal bleeding, and one patient survived >12 months after AIC. One patient died of systemic bone metastasis 12 months after procedure.

Currently, four patients are alive. One patient is receiving postoperative radiotherapy, has survived for 36 months, and is consuming a liquid diet. Two patients have received immune-targeted therapy: one patient has survived for >36 months and the other patient has survived for 24 months without dysphagia/dyspnea symptoms. The other patient has survived the longest (>42 months) without dyspnea symptoms. For these four cases, 1-year survival is 46.0%, and the median duration of survival is 8.5 months.

## Discussion

Recurrence of an esophagogastric anastomotic stricture is more likely to occur <2 years after esophagectomy (3, 18, 19) and it may be related to metastasis to lymph nodes and intramural metastasis of esophageal cancer (20). Rasihashemi and colleagues (21) reported that the anastomotic type is related to benign stenosis, but not to recurrent stenosis. The duration of survival of patients with recurrence of esophageal cancer after surgery is very short. Yuichiro and colleagues (22) reported that the median duration of survival of patients suffering from esophageal cancer with local recurrence after surgery was ~178 days. According to Butterer and colleagues, the median overall survival of patients with local recurrence was 4.9 months, that of patients with distant metastasis was 2.9 months, and that of all patients was 3.2 months (23).

Radiotherapy is a common method for treatment of local recurrence of esophageal cancer after surgery. However, the optimal radiation dose of radiotherapy is controversial. High-dose radiotherapy can lead to esophageal strictures, and the effect of radiotherapy alone is poor (24–27). Reports (8, 28) have suggested that surgical lymphadenectomy combined with radiotherapy and chemotherapy can improve the chance of survival of patients with local recurrence of esophageal cancer after surgery, but it can accelerate the risk of metastasis of tumor cells to organs.

Stent placement into the esophagus has been used widely because of its rapid improvement of dysphagia, and is the most common treatment for NEAS (1, 29). Pinto and colleagues (30) reported that in the palliative treatment of patients with advanced esophageal cancer, the patency duration of an esophageal stent in 42 patients was 236 days, and that the clinical symptoms of all patients were improved. However, implantation of a simple stent into the esophagus only temporarily solves the problem of an esophagogastric anastomotic stricture and does not treat the primary disease. In a study by Bi and coworkers (31), 22 patients with a malignant esophageal stricture underwent implantation of a segmental radioactive metal stent into the esophagus and, compared with patients implanted with a traditional esophageal metal stent, survived longer. Tinusz and collaborators (32) reported that, after implantation of a radioactive stent into the esophagus in 1177 patients, compared with the traditional esophageal stent, it had more advantages for reversing dysphagia and prolonging the life expectancy of patients. However, patients with a neoplastic esophagogastric anastomosis in the neck or arch have a short residual esophagus or angle between the esophagus and remnant stomach. Stent implantation into the esophagus is prone to complications such as displacement, perforation and bleeding (33, 34). Das and colleagues (35) reported that in 357 patients with dysphagia due to esophageal malignancy, the prevalence of stent displacement was 15.4%, and the prevalence of stricture recurrence caused by stent blockage by a malignant tumor was 12.6%. Therefore, if rapid shrinkage of the tumor and reduction of compression can be achieved, then stent implantation in the esophagus for patients with a malignant esophagogastric anastomotic stricture can be avoided (along with avoidance of the complications caused by esophageal stents).

AIC is based on highly selective local infusion of a chemotherapy drug through percutaneous arterial puncture. AIC has been used to treat cancer of the liver, colorectum and esophagus (12, 13, 36, 37). Yin et al. (12) reported that 75 cases of advanced esophageal cancer underwent 1–3 cycles of AIC, and the total effective rate (CR + PR) was 94.7%, and dysphagia was significantly improved. Compared with systemic chemotherapy, AIC can increase the concentration of the anticancer drug in the tumor and reduce the risk of side-effects (38, 39). These actions can shrink the tumor rapidly, thereby relieving/alleviating airway invasion.

In the present study, 50 patients with dysphagia improved after AIC. Simultaneously, 38 patients with different degrees of dyspnea improved after AIC. Fifty patients completed 1–3 courses of AIC, and the ORR was 72.0%, and DCR was 100%. The main complication of AIC was postoperative formation of esophageal fistulae. One patient had a fistula in the neck portion of the esophagus neck after AIC and was clinically evaluated as a PR. Considering the rapid reduction in tumor size after surgery, the surrounding healthy tissue was not repaired sufficiently, and a nutrition tube was inserted. The surrounding healthy tissue healed 3 weeks later.

In the present study, AIC for treatment of NEAS after esophagectomy achieved good clinical results. The arteries of esophageal carcinomas are variable and complex but, in the normal anatomical structure, different segments of the esophagus have their own relatively fixed feeding vessels. However, for patients with postoperative recurrence, the feeding arteries of the tumor change due to alterations in their original anatomical position. Only by finding all the feeding arteries of the tumor can the best clinical effect of AIC be achieved. Application of a microcatheter in AIC for esophageal tumors can effectively: (i) improve the local concentration of anticancer drugs: (ii) reduce damage to non-target blood vessels caused by chemotherapy drugs; (iii) prevent serious complications (e.g., malperfusion). If the tumor reaches the descending stage after AIC, radiotherapy and targeted therapy should be carried out when the physical condition permits, so as to strengthen the local control of tumor and improve the curative effect and long-term survival rate of patients.

The main limitations of our study were that it was a single-center retrospective analysis with selection biases. We hope to conduct a multi-center, large-cohort prospective study in the near future.

AIC is safe and efficacious for NEAS treatment after esophagectomy. AIC should be considered for clinical application against NEAS.

## Data Availability Statement

The original contributions presented in the study are included in the article/supplementary material. Further inquiries can be directed to the corresponding author.

## Ethics Statement

The studies involving human participants were reviewed and approved by Ethics Committee of the First Affiliated Hospital of Zhengzhou University. The patients/participants provided their written informed consent to participate in this study. Written informed consent was obtained from the individual(s) for the publication of any potentially identifiable images or data included in this article.

## Author Contributions

PX and GW contributed in the study design, data collection, data analysis, data interpretation, literature search, and writing of the article. MY contributed in the literature search and writing of the article. WH contributed in the data collection, analysis, and interpretation. YM, CL, and ZL contributed in the data analysis and data interpretation. XL and SW contributed in the study design and data interpretation. All authors contributed to the article and approved the submitted version.

## Funding

This study was supported by the Provincial Ministry Co-Construction Project from the Medical Scientific and Technological Research Program of Henan Province (Grant no. SB201901035).

## Conflict of Interest

The authors declare that the research was conducted in the absence of any commercial or financial relationships that could be construed as a potential conflict of interest.
